# Autumn migratory orientation and route choice in early and late dunlins *Calidris alpina* captured at a stopover site in Alaska

**DOI:** 10.1242/bio.058655

**Published:** 2021-04-27

**Authors:** Susanne Åkesson, Johanna Grönroos, Giuseppe Bianco

**Affiliations:** 1 Department of Biology, Centre for Animal Movement Research, Lund University, Ecology Building, 22362 Lund, Sweden; 2 Department of Environmental Science and Bioscience, Kristianstad University, 29188 Kristianstad, Sweden

**Keywords:** Bird migration, Magnetic compass, Orientation, Route simulation, Sun compass

## Abstract

We investigated the migratory orientation of early and late captured dunlins, *Calidris alpina*, by recording their migratory activity in circular orientation cages during autumn at a staging site in southwest Alaska and performed route simulations to the wintering areas. Two races of dunlins breeding in Alaska have different wintering grounds in North America (Pacific Northwest), and East Asia. Dunlins caught early in autumn (presumably *Calidris alpina* *pacifica*) oriented towards their wintering areas (east-southeast; ESE) supporting the idea that they migrate nonstop over the Gulf of Alaska to the Pacific Northwest. We found no difference in orientation between adult and juveniles, nor between fat and lean birds or under clear and overcast skies demonstrating that age, energetic status and cloud cover did not affect the dunlins’ migratory orientation. Later in autumn, we recorded orientation responses towards south-southwest suggesting arrival of the northern subspecies *Calidris alpina* *arcticola* at our site. Route simulations revealed multiple compass mechanisms were compatible with the initial direction of early dunlins wintering in the Pacific Northwest, and for late dunlins migrating to East Asia. Future high-resolution tracking would reveal routes, stopover use including local movements and possible course shifts during migration from Alaska to wintering sites on both sides of the north Pacific Ocean.

## Abstract (precis)

**Summary:** Orientation experiments with dunlins captured in Alaska during autumn migration confirm orientation to distant wintering areas. Route simulations revealed multiple compass mechanisms were compatible with the initial direction of early dunlins wintering in the Pacific Northwest, and for dunlins migrating to East Asia.

## INTRODUCTION

Many arctic breeding waders fly impressively long distances between their summer and winter quarters. Overall migratory distances exceeding 10,000 km are not uncommon, and some species even make single non-stop flights that cover several thousands of kilometres (e.g. Morrison, 1984; Pienkowski and Evans, 1984; Gill et al., 2009; Klaassen et al., 2011; Johnson et al., 2015; Lisovski et al., 2016). Performing such long migrations including non-stop flights requires precise orientation mechanisms. Birds have been shown to use a complex array of directional cues, such as the position of the sun, the skylight polarization patterns, the rotation of the starry sky, and directional information from the geomagnetic field, to select a seasonally appropriate compass course during migration (for reviews see, Emlen, 1975a; Able, 1980; Wiltschko and Wiltschko, 1995; Åkesson et al., 2014). Still we have limited understanding of which compass systems birds use during migratory flights (cf. Åkesson and Bianco, 2016, 2017).

Birds may follow several alternative routes on migration (e.g. Gudmundsson and Alerstam, 1998; Åkesson and Bianco, 2016, 2017; Muheim et al., 2003, 2018). The orthodrome (great circle) is the shortest route between two points on the Earth's surface, but requires continuous course changes as the birds’ moves across longitudes (Snyder, 1993). The loxodrome (rhumb line) route usually represents a longer distance, but may be convenient from an orientation point of view because it is associated with a constant compass course (Snyder, 1993), and may be guided by celestial cues providing information of geographic north. Birds relying on a time-compensated sun compass without compensating for the time shift during flight may follow approximate orthodrome routes (Alerstam and Pettersson, 1991), while geomagnetic loxodromic and magnetoclinic routes (Kiepenheuer, 1984) are selected relative to geomagnetic north and the angle of inclination, respectively.

Our knowledge of the mechanisms of compass orientation originates mainly from orientation cage experiments performed with nocturnally migrating passerines (Emlen, 1975a; Able, 1980; Wiltschko and Wiltschko, 1995; Åkesson et al., 2014). Even though waders have been shown to be suitable for orientation cage experiments, only a limited number of studies have been performed (e.g. Sauer, 1963; Sandberg and Gudmundsson, 1996; Gudmundsson and Sandberg, 2000; Grönroos et al., 2010; Hua et al., 2017, Vanni et al., 2017).

**Fig. 1.**
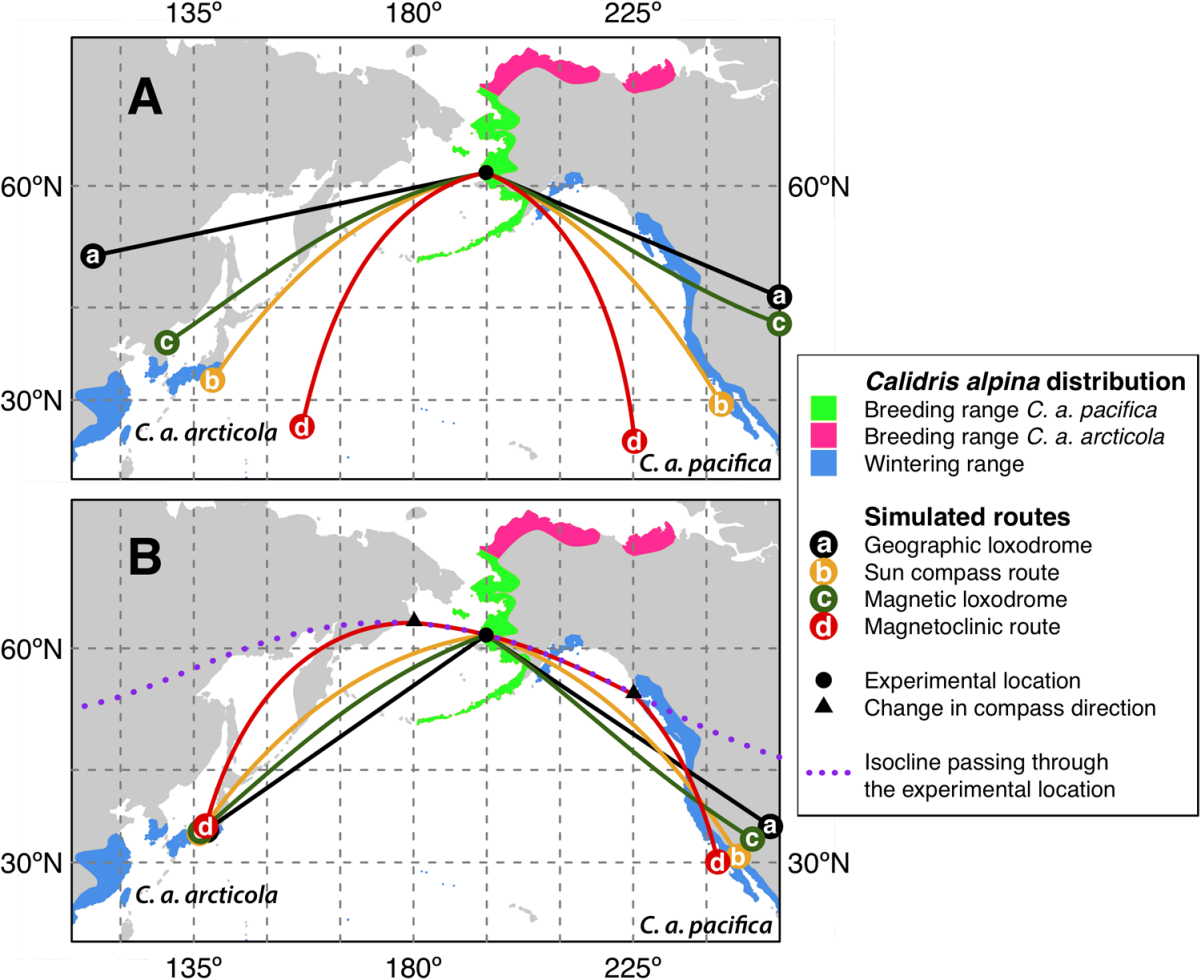
**Maps of breeding and wintering ranges of the two different races of dunlins (*Calidris alpina*) breeding in Alaska and simulated routes generated by different compass mechanisms.** Species range is from BirdLife International and Handbook of the Birds of the World (2017, Version 7.0 and available at http://datazone.birdlife.org/species/requestdis). Separation of breeding ranges of *C. a. pacifica* and *C. a. arcticola* are extrapolated from genetic analysis in Wennerberg and Bensch (unpublished data). (A) Simulated routes from the study site in the Yukon-Kuskokwim Delta in SW Alaska using the same initial geographical direction for all compass mechanisms. (B) Simulated routes with initial direction of each compass mechanism selected to be compatible with the expected wintering range of the two dunlin races. Routes are 5000 km (a and c in panel A extend c.a. 500 km further outside map edge and the rest of the route is not plotted because it has already crossed the wintering area). Maps are drawn in Mercator projection with 15° grid.

Two races of dunlin (*Calidris alpina*) breed in Alaska (Fernández et al., 2008). The most numerous of the two races *Calidris alpina* *pacifica* breeds in coastal areas in western Alaska including the Alaska Peninsula, and winters along the west coast of North America from southern Canada to Mexico, while *Calidris alpina* *arcticola* breeds in northern Alaska and winters in coastal East Asia (Warnock and Gill, 1996; Fig. 1). The two races are difficult to separate from each other, but if you have adult birds with known sex it is possible to separate them by bill length (Warnock and Gill, 1996). During autumn migration *C. a. pacifica* stage in western and southern Alaska and probably fly nonstop over the Gulf of Alaska to the Pacific Northwest and the wintering areas further south (S) along the North American west coast (Warnock and Gill, 1996; Warnock et al., 2004; Taylor et al., 2011). This nonstop transoceanic flight occurs mainly during end of September to October in association with predictable weather systems favourable for migration across the Gulf of Alaska (Warnock and Gill, 1996). There seem to be two different populations of *C. a. pacifica*, one that breed and stage in the Yukon-Kuskokwim Delta and winter in the Pacific Northwest while the other population breed and stage on the Alaska Peninsula and winter further S in California (Warnock and Gill, 1996). Some birds of the race *C. a. arcticola* may leave northern Alaskan breeding grounds directly for wintering areas in East Asia (Norton, 1971), but most of these dunlins are thought to continue S to Yukon-Kuskokwim Delta where they mix with *C. a. pacifica* before migrating to Asia in September or October (Warnock and Gill, 1996; Warnock et al., 2013; Taylor et al., 2011). Dunlins of the race *C. a. arcticola*, arrive on the Yukon-Kuskokwim Delta from mid-August and onward (Taylor et al., 2011). The preferred route from breeding to wintering grounds is unknown for *C. a. arcticola*, but the possible use of a coastal route along western Bering Sea and Sea of Okhotsk have been proposed (Warnock and Gill, 1996; Fernández et al., 2008).

The aim of our study was to examine the migratory orientation of early and late dunlins from two different races (*C. a. pacifica* and *C. a. arcticola*) captured during autumn migration at a staging site in Alaska and to evaluate the differential route choices. We used the orientation data to evaluate alternative compass routes by numerical simulations, which the birds might use during their flights. We also performed three additional simulations where we examined which alternative compass route may lead to predicted destination areas in Asia and North America. We examined if fat content and age of the dunlins influenced their directional choices recorded in the orientation cages, where reverse orientation was predicted for lean birds as compared to fat birds preferring to depart in the expected migratory direction.

## RESULTS

**Fig. 2.**
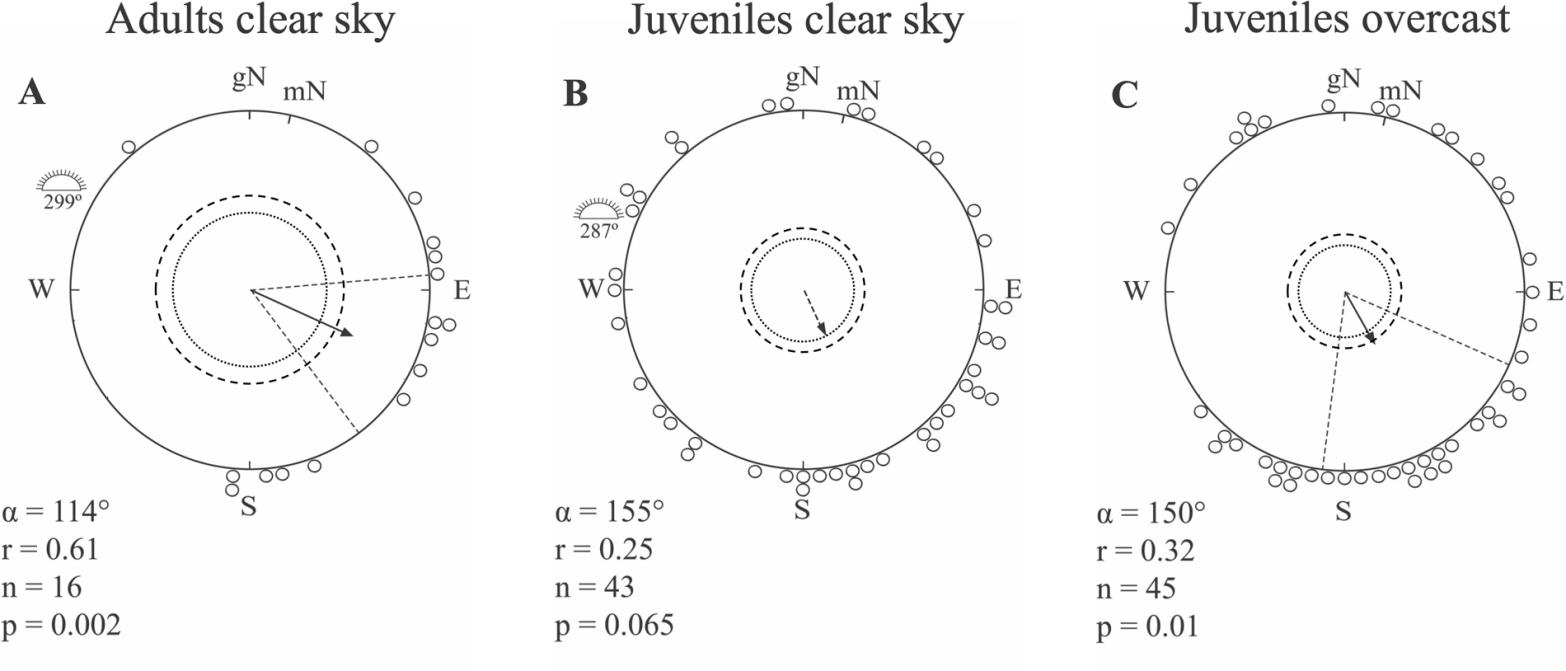
**Orientation of migratory adult and juvenile dunlins under clear and overcast skies in the Yukon-Kuskokwim Delta, SW Alaska in autumn.** Adults were caught between 8 and 11 August while juveniles were caught both between 8 and 11 August and between 4 and 9 September. Each circle at the periphery of the diagrams represents the mean direction of one individual bird during one experimental hour (one individual is only represented once in each diagram). Individual mean headings are shown in relation to geographic North (gN) and magnetic North (mN). Experiments were conducted in the local geomagnetic field. The sun indicates the mean position of the setting sun along the horizon in the middle of the experimental period. The mean vector (*α*) of each sample is illustrated by an arrow surrounded by the 95% confidence interval (dotted lines) in cases with statistical significance. Arrow lengths are proportional to the mean vector lengths (*r*) and are drawn relative to the radius of the circles (radius=1). Significance levels at 5% and 1% are reported as dotted and dashed circular lines, respectively, and the value of the Rayleigh test (P) according to Batschelet (1981). Circular statistics data for A–C provided in Supplementary Table S1.

Adult dunlins tested in the local geomagnetic field under clear skies selected a significant mean direction towards geographic ESE (*α*=114°, *r*=0.61, *n*=16, *P*<0.002; Fig. 2A), which is significantly different from the sun azimuth in the middle of the experimental hour (mean sun direction: *α*=299°; 95% confidence interval: *P*<0.05). Adult birds were caught early in the migratory season (11 August), and were most probably birds from the race *C. a. pacifica*. Their mean orientation was very concentrated and coincided well with the expected migratory direction of that race towards ESE (Fig. 2A). All juveniles from both capture periods tested under clear skies in the local geomagnetic field, showed larger scatter in their directional preferences as compared to the adults, and their mean orientation was not significantly different from random (*α*=155°, *r*=0.25, *n*=43, *P*=0.065; Fig. 2B). However, juvenile dunlins tested under natural and simulated overcast conditions selected a significant mean direction towards geographic SSE (*α*=150°, *r*=0.32, *n*=45, *P*=0.01; Fig. 2C).

**Fig. 3.**
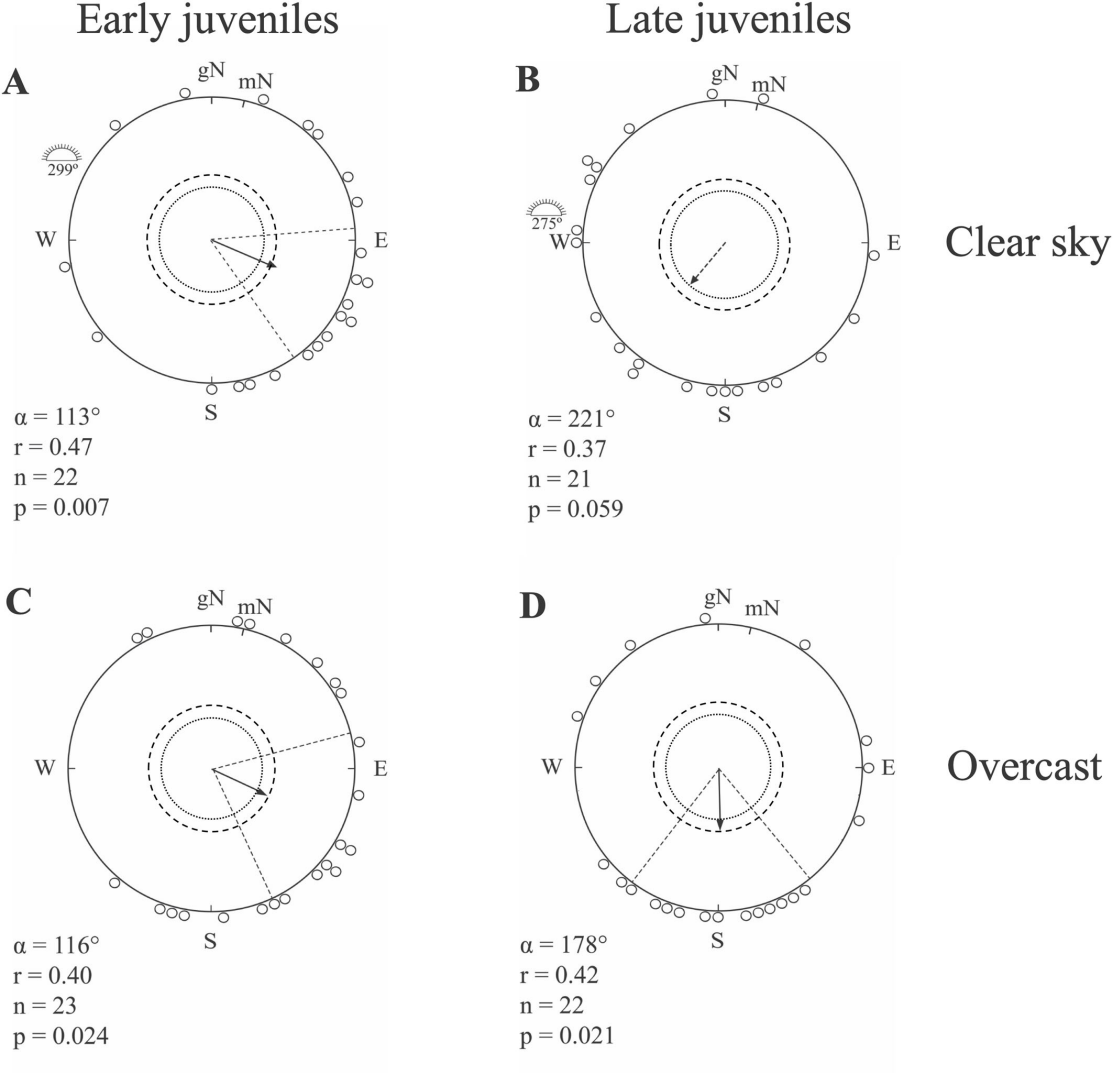
**Orientation of migratory early and late caught juvenile dunlins under clear and overcast skies in the Yukon-Kuskokwim Delta, SW Alaska in autumn.** Early juveniles were caught between 9 and 11 August while late juveniles were caught between 4 and 9 September. For further information see Fig. 2. Circular statistics data for A–D provided in Supplementary Table S2.

Since we were unable to identify the race of the juvenile dunlins in the hand, and as they were captured both early and late in the autumn migratory season, we separated the juvenile dunlins into two different groups depending on when during the season they were caught. By doing so we expected juvenile dunlins caught early (9–11 August) to predominantly belong to the race *C. a. pacifica.* When tested in the local geomagnetic field under clear skies, this group of juvenile dunlins selected a mean orientation towards geographic east-southeast (ESE) (*α*=113°, *r*=0.47, *n*=22, *P*=0.007; Fig. 3A). This direction was significantly different from the sun azimuth in the middle of the experimental hour (mean sun direction: *α*=299°; 95% confidence interval: *P*<0.05), but not different from the mean direction of adult dunlins caught during the same period (Mardia's one-way classification test: *F*_1,36_=0.0015, *P*=0.97). Furthermore, we found that there was no difference in scatter between tests with adults and early caught juveniles under clear skies (Mardia's test for homogeneity of concentration parameters: *t*_36_=0.72, *P*>0.05). We are aware that juvenile dunlins caught later in the season (4–9 September), may belong to either *C. a. pacifica* or *C. a. arcticola*, but we expected a higher input of the latter subspecies at this time due to their later arrival to the stopover sites. The juvenile birds from this late period selected a mean orientation towards geographic southwest (SW) under clear skies, which was just not significant (*α*=221°, *r*=0.37, *n*=21, *P*=0.059; Fig. 3B). The mean direction of late caught juvenile dunlins differed significantly from both early caught adult and juvenile dunlins (Watson's *U*^2^-test: *U*^2^=0.28 and 0.29, respectively, *P*<0.01 in both cases). Early caught juveniles tested under natural and simulated overcast conditions showed a significant mean direction towards geographic ESE (*α*=116°, *r*=0.40, *n*=23, *P*=0.024; Fig. 3C), not different from the mean orientation recorded under clear skies (Mardia's one-way classification test: *F*_1,43_=0.0113, *P*=0.92). There was no difference in scatter between clear sky and overcast experiments in early caught juveniles (Mardia's test for homogeneity of concentration parameters: *t*_43_=−0.36, *P*>0.05). Dunlins caught late in the migratory season tested under natural and simulated overcast conditions showed a significant mean direction towards S (*α*=178°, *r*=0.42, *n*=22, *P*=0.021; Fig. 3D), and this orientation was not different from the mean orientation recorded under clear skies (Watson's *U*^2^-test: *U*^2^=0.053, *P*>0.5).

We did not find any difference in mean orientation between fat and lean dunlins caught early for each experimental category (see Table 2, adult clear sky: Mardia's one-way classification test: *F*_1,14_=0.14, *P*>0.5; juvenile clear sky: Watson's *U*^2^-test: *U*^2^=0.087, *P*>0.2; juvenile overcast: *U*^2^=0.069, *P*>0.5). Furthermore, there was no difference in scatter in orientation between lean and fat adult dunlins (Mardia's test for homogeneity of concentration parameters: *t*_14_=−1.15, *P*>0.05).

A Chi-square test revealed a significant difference in inactivity between the different experimental categories (Chi-square test: *χ*^2^_10_=25.1, *P*=0.005, see Table 1). To investigate this further, we looked into whether there were any differences in numbers of active and inactive juvenile dunlins between tests under clear skies and overcast, respectively. We found that birds were significantly more likely to become inactive under overcast than under clear skies (44 versus 17%, Chi-square test: *χ*^2^_2_=13.4, *P*=0.0012). We also found that early caught dunlins were more inactive than late caught birds (42 versus 26%, Chi-square test: *χ*^2^_1_=4.4, *P*=0.035). However, there were no differences in activity between fat and lean birds (Chi-square test: *χ*^2^_1_=3.0, *P*>0.05).

**Table 1.**
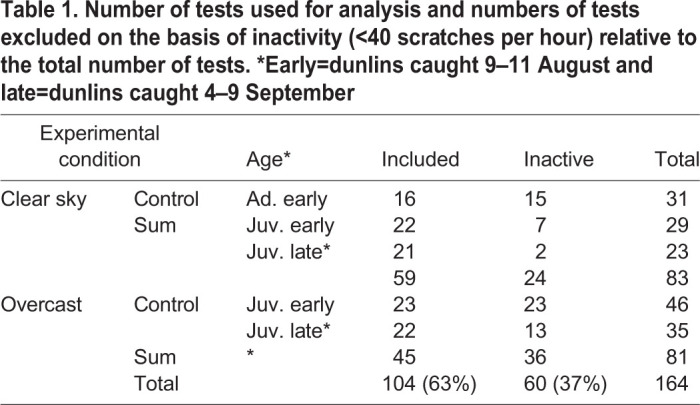
Number of tests used for analysis and numbers of tests excluded on the basis of inactivity (<40 scratches per hour) relative to the total number of tests. *Early=dunlins caught 9–11 August and late=dunlins caught 4–9 September

**Table 2.**
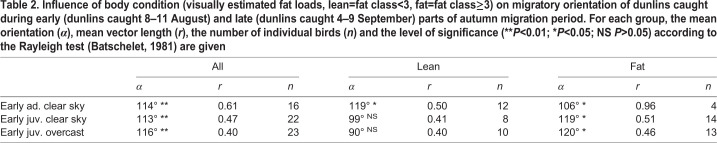
Influence of body condition (visually estimated fat loads, lean=fat class<3, fat=fat class≥3) on migratory orientation of dunlins caught during early (dunlins caught 8–11 August) and late (dunlins caught 4–9 September) parts of autumn migration period. For each group, the mean orientation (*α*), mean vector length (*r*), the number of individual birds (*n*) and the level of significance (**P<0.01; *P<0.05; NS *P*>0.05) according to the Rayleigh test (Batschelet, 1981) are given

The first set of simulations used the same initial geographic direction for all compasses. We used *α*=113° for *C. a. pacifica* and *α*=258° for *C. a. arcticola* (Fig. 1A). For *C. a. pacifica* the direction was the mean of early juveniles under clear sky (Fig. 3A), and showed that geographic and magnetic loxodrome reached the wintering range in Pacific Northwest at more northerly latitude than the sun compass, but the magnetoclinic route was not successful being far distant from the Pacific Northwest costal area (Fig. 1A). For *C. a. arcticola* we used the direction toward the SW that would minimise the average distance of the final destination of all routes to the northern extension of the species’ wintering range. Such initial direction choice for *C. a. arcticola* produced almost symmetrical geometries compared to *C. a. pacifica* routes with a relative wide spread of the four possible routes considered. Similarly, to the simulations for *C. a. pacifica*, geographic and magnetic loxodrome for *C. a. arcticola* resulted in more northerly routes than the sun compass and the magnetoclinic routes (Fig. 1A).

The second set of simulations with specific departure directions for each compass produced alternative scenarios for both dunlin races. For *C. a. pacifica* the routes crossed a large section of the wintering range. However, since the Pacific Northwest wintering range is quite broad from north to south, many alternative solutions could be compatible (Fig. 1B). The range of directions presented in Fig. 1B was from *α*=104° of the magnetoclinic route to *α*=124° of the geographic and magnetic loxodromes, and all resultant routes were within the confidence interval of early juveniles’ orientation under both clear (clear sky 95% confidence interval: ±36°, *P*<0.05 for all; Fig. 3A) and overcast skies (overcast 95% CI: ±45°; *P*<0.05 for all; Fig. 3C). For *C. a. arcticola* we set the destination goal to south Japan and found that geographic loxodrome, magnetic loxodrome and sun compass initial direction where all in a SW direction (*α*=235°, 250° and 262°, respectively), whereas the magnetoclinic was the only one with a northwest (NW) departure (*α*=285°; Fig. 1B). The mean orientation recorded for the late juveniles under clear sky conditions were not significantly different from random (*P*=0.059; Fig. 3B).

An additional note to the magnetoclinic route is that a change in route of the compass direction must occur for both dunlin races whether they winter in the Pacific Northwest or in East Asia. In our simulations we assumed that birds were following the isocline (i.e. the isoline following the same geomagnetic inclination), passing the departure location until crossing the coastline (black triangles in Fig. 1B) and then following the chosen magnetoclinic course based on a fix apparent magnetic inclination. This extra assumption is still compatible with a single inclination compass mechanism (Kiepenheuer, 1984, and discussion below) and interestingly produced the route geometry closest to the coastline for both scenarios evaluated. In particular, *C. a. pacifica* hit the winter distribution of the Northwest Pacific at its northernmost extension and crossed the entire wintering range till South California (Fig. 1B). On the other hand, *C. a. arcticola* was able to cross the Bering Sea and Sea of Okhotsk as suggested by Warnock and Gill (1996) with the same mechanism. It should be noted that, in both scenarios, the change in magnetoclinic direction can occur at different locations producing different geometries (either closer or far from continental coastlines), but the initial direction will always be the same and specifically the one that will follow the isocline (cf. Fig. 1B) for the departure location and time considered. Thus, dunlins that will migrate to the Northwest Pacific will always depart in the southeast (SE) direction from our study site and dunlins migrating to East Asia will always depart in the NW direction (Fig. 1B).

## DISCUSSION

Both adult and juvenile dunlins caught early in the migratory season (belonging to the race *C. a. pacifica*, see above) oriented towards their wintering areas in the Pacific Northwest (Warnock and Gill, 1996; Fig. 1) when recorded in orientation cages. If the dunlins were to follow a local coastal route to their wintering areas they would orient towards SW or S, but instead the migratory directions recorded in our study were oriented towards ESE, supporting the idea that these birds make direct flights from staging areas in Alaska initially crossing land and leading them to their wintering areas in the Pacific Northwest (Warnock and Gill, 1996). Multiple compass mechanisms were also compatible with direct flights from SW Alaska to the Pacific Northwest costal area. These non-stop transoceanic flights have been shown to occur in association with predictable weather systems generating tailwinds favourable for migration across the Gulf of Alaska (Warnock and Gill, 1996). Several other bird species (e.g. Gill et al., 1997, 2009, 2014) have evolved this wind-sensitive migration strategy associated with the Aleutian low pressure system (Gill et al., 2005), but also in other regions (e.g. Gudmundsson, 1993; Åkesson and Hedenström, 2000; Åkesson et al., 2002, 2016; Conklin and Battley, 2011; Ma et al., 2011; Sjöberg et al., 2015).

Several Arctic breeding wader species have been suggested to migrate along orthodromic routes between Siberia and North America (Alerstam and Gudmundsson, 1999; Alerstam et al., 2001; 2007; Grönroos et al., 2010), but from our results, it was difficult to tell which of the routes the dunlins were intending to follow. There are indications that waders follow both loxodromic (Alerstam et al., 1990; Gudmundsson, 1994) and orthodromic routes (Alerstam and Gudmundsson, 1999; Alerstam et al., 2001; 2007) during migration depending on different environmental conditions such as latitude, topography, wind and weather conditions, food availability and stopover sites associated with different flight paths (Alerstam, 2001). In our route simulations, we did not find support for compass mechanisms generating routes similar to loxodromes (i.e. geographic loxodrome and magnetic loxodrome) or guiding birds along more curved routes approximating orthodromes (i.e. sun compass and magnetoclinic route). All routes produced using either celestial or magnetic compasses, were slightly different in geometry, but were also all compatible with the birds’ final two destinations. Our data suggest that a strategy using either of the two compasses or a combination of both will lead the waders to their known destinations, and that this strategy may have been part of the range expansion process, as the same mechanism will lead the birds along similar routes in both directions (Åkesson and Bianco, 2017; Sokolovskis et al., 2019).

Early caught dunlins from the most southern subspecies *C. a. pacifica* were tested in August, which is a few weeks before the peak migration period (Warnock and Gill, 1996), and yet they showed orientation towards their wintering grounds suggesting high motivation to move in this direction at an early stage of the season. Still we noted that a higher proportion of these birds were inactive compared to dunlins caught and tested later in the migratory season. We also found that the scatter in orientation was rather high, especially for juvenile birds compared to the orientation recorded for the experienced adult birds. This is not surprising as it is often observed in orientation cage studies that the directional preferences in juvenile birds are more scattered compared to adults (e.g. Åkesson et al., 2001, 2005, 2021). The reason may be related to the need to have access to more information and see a larger part of the sky to find a meaningful migratory direction, which may be more important in juveniles than in adults (Åkesson et al., 2021). Under clear skies, we furthermore found a higher number of juvenile individuals expressing orientation directions towards west coinciding with the sunset position than under overcast (Fig. 3), and the reason could be a higher attraction towards the brightest part of the sky in juvenile than adults tested in orientation cages (Åkesson et al., 2021). It could also be that in the juvenile population, there are a larger number of individuals with slightly deviating preferred directions, which may not be successful when migration is initiated as compared to the adult group (Sergio et al., 2014; Åkesson et al., 2021).

Some of the dunlins in our study might be locally breeding birds, but they can also be migrants from breeding areas outside the delta (Handel and Gill, 1992). Post-breeding dunlins occur in the area from July until early October, since the Yukon-Kuskokwim Delta provide extremely abundant food resources, and waders fuelling for migration are recruited from extensive inland and coastal areas to the coastal foraging sites (e.g. Gill and Handel, 1990; Gill and Senner, 1996; Lindström et al., 2011). Already at the test occasion in August, our dunlins were migratory active showing meaningful migratory directions in the cages suggesting they were at that time expressing behaviours in line with their inherited migration program.

We found no difference in the ability to orient between juvenile and adult dunlins, and furthermore there was no difference among juveniles between clear and overcast skies, suggesting a good ability to find appropriate migratory directions even in situations with restricted access to visual compass cues (overcast; Åkesson et al., 2014, 2015). These results indicate that juvenile dunlins did not have any difficulties in choosing the appropriate migratory direction under overcast conditions when mainly the magnetic compass were available to them and they could not primarily rely on their sun or stellar compasses (Able, 1980; Åkesson et al., 2014). From an orientation point of view, we would not expect a difference in orientation between clear sky and overcast conditions, since migratory birds are expected to calibrate all of their magnetic and celestial compasses, i.e. bring them in conformity with a common reference during a stopover (Muheim et al., 2006, cf. Åkesson et al., 2015). It has been shown that migratory waders are less motivated to depart from stopover sites under overcast skies (e.g. Piersma et al., 1990), and the majority of orientation studies on waders have shown that the birds were either inactive, disoriented or showing a bimodal distribution of preferred headings when tested under overcast conditions (Sauer, 1963; Sandberg and Gudmundsson, 1996; Gudmundsson and Sandberg, 2000). A high proportion of the dunlins in our study were inactive under overcast skies, which has also been shown for passerines (e.g. Åkesson, 1993, 1994), but when active they showed orientation in agreement with the expected migratory direction. It suggests that they may have used a combined compass strategy, which include both celestial and magnetic information, for orientation. In contrast to the findings above, orientation experiments with juvenile sharp-tailed sandpipers, *Calidris acuminata*, in Alaska showed that the birds were as active under overcast as under clear skies and they displayed a significant mean orientation indicating that they did not have a problem finding a meaningful orientation under overcast skies (Grönroos et al., 2010). Thus, in two species of waders departing from staging sites in southwestern Alaska, we found high migratory activity and significant mean orientation under overcast skies, when the degree of skylight polarization is reduced but can still be perceived from a naturally overcast sky near the horizon (Hegedüs et al., 2007).

We did not find any difference in activity, migratory orientation or concentration between fat and lean dunlins. Many studies have shown that the amount of stored fat is an important predictor for selection of migratory directions in birds, especially when migrants are confronted with an ecological barrier (e.g. Sandberg, 1994; Åkesson et al., 1996; Bäckman et al., 1997; Sandberg et al., 2002; Deutschlander and Muheim, 2009). Individuals that carry insufficient fat stores will either suppress migratory activity (i.e. stay on their current location if foraging conditions are favourable) or, if feeding opportunities are restricted, reorient in search for more profitable habitats (Sandberg, 2003). In inland areas, reverse orientation is not expressed at all to the same degree (Åkesson, 1999). Given the migratory behaviour of *C. a. pacifica* we expected that lean birds would be less active or show initial migration towards the coast in search of more profitable habitats, but instead they seem to show their innate migratory direction towards the wintering grounds regardless of energetic status. This difference in behaviour between songbirds and waders might be a result of where the stopover sites are located relative to the barrier. In songbirds, the preferred habitat most often occur inland, away from the coast (Alerstam, 1978), while in waders the best foraging zone are found at the coast itself (Gill et al., 2013). Therefore, it might be a difference in how waders compared to passerines respond when confronted with the coast depending on where they are caught and tested, where waders might be expected to orient towards the coast to find good foraging conditions, while songbirds instead may move away from the coast to more protected and favourable inland sites (e.g. Åkesson et al., 1996, Buler et al., 2007).

Our late caught dunlins oriented more to the SW and S compared to early-caught individuals, which might be the result of a mixture of dunlins from the two different races *C. a. pacifica* and *C. a. arcticola*, showing different orientation preferences during the late part of the season. However, if this was the case we would expect a more bimodal orientation towards ESE (*C. a. pacifica*) and west-southwest (WSW) (*C. a. arcticola*) than was recorded (a major part of the birds expressed southerly orientation), and therefore the southerly direction shown under overcast is a bit surprising. However, we recorded a slightly more southerly orientation also under clear sky conditions for this late group (just not significantly different from random, *P*=0.059). Moreover, according to our simulations, the magnetoclinic route predicted a NW direction for this group. We did not expect this result before running the simulations since this outcome is the consequence of the features of the local geomagnetic field (see discussion below). We notice a fraction of the birds expressed preferred NW orientation in both juvenile groups (Fig. 3), which suggest the possibility that a number of juveniles of *C. a. arcticola* were present in our tested group and that the inclination of the Earth's magnetic field might have been used to find a meaningful direction. Furthermore, by following the southerly direction recorded under overcast for a long stretch of time, the dunlins are likely to end up too far south and in the open waters of the Pacific Ocean, which is not a likely scenario. Following a southerly course during the initial part of their sea-crossing, the dunlins will pass the Aleutians stretching across the ocean south of Alaska, and whether they will change the course or even land at more southern island stopover sites remains to be investigated.

Our route simulations did not support any specific vector-navigation mechanisms. Different compasses could produce realistic routes for both dunlin races to their respective winter destinations. Alternative initial directions would also be compatible with the extended wintering range of this species especially in the Pacific Northwest. Additional assumptions for the sun compass, such as re-calibration at different time of the day or excluding compensation for apparent motion of the sun (Muheim et al., 2018) or adding a partial time compensation (Sokolovskis et al., 2019), would not change the above overall conclusion for our specific scenario. Better predictions could be made in the future, when routes and more defined wintering areas could be obtained by tracking individual birds.

Only for the magnetoclinic route we could predict a fixed departure direction under the assumption that birds will start following the isocline at the departure location, and later they will change the compass direction that will bring the birds at more southerly latitudes. This possibility was originally suggested by Kiepenheuer (1984) for birds migrating from the high arctic tundra in northeast Russia to wintering areas in Africa. Such idea has been shown to be feasible for the northern wheatear *Oenanthe oenanthe* breeding in Alaska (Åkesson and Bianco, 2017), and more recently for willow warblers *Phylloscopus trochilus yakutensis* breeding in northeast Siberia (Sokolovskis et al., 2019). However, our example here is an additional case, for which this two-step magnetoclinic route will be necessary for a migratory system additional to those mentioned by Kiepenheuer (1984). It is still necessary, however, to prove that an inclination compass following the apparent angle of inclination could be use by birds while in flight (cf. Alerstam, 1987).

In this study, we have shown that cage experiments recording migratory activity in individual birds reveal differences in preferred orientation that correspond to migration routes that lead the two subspecies of dunlins captured at the same site, to non-breeding areas in different continents. In the future, the use of high-resolution tracking technology in combination with route simulations will better reveal if a specific compass alone can explain the routes selected, or if a more complex navigation strategy are used by this long-distance migratory wader to reach the non-breeding destinations potentially including course shifts.

## MATERIALS AND METHODS

### Study site and experimental procedure

Dunlins were captured during autumn migration in the Yukon-Kuskokwim Delta in SW Alaska, a highly important staging and stopover area for large numbers of migrating tundra birds (e.g. Gill and Handel, 1990; Gill and Senner, 1996; Gill et al., 2013). We carried out the orientation cage experiments at Kanaryarmiut Field Station (61°21′N, 165°08′W) on the Yukon-Kuskokwim Delta, SW Alaska, from early August until late September 2005 (Fig. 1) during the Swedish research expedition Beringia 2005 (Rickberg, 2006). Kanaryarmiut Field Station is located inland approximately 25 km from the coast near the Aphrewn River and the site consists of upland heath tundra (Nebel and McCaffery, 2003). Dunlins (*N*=164) were caught in the area using ‘Ottenby’ walk-in traps (Lindström et al., 2005) between 8–11 August (capturing mainly birds belonging to the race *C. a. pacifica*), and between 4–9 September (likely capturing birds belonging to both races; *C. a. pacifica* and *C. a. arcticola*; Taylor et al., 2011, Gill et al., 2013). The captures of experimental birds were timed to occasions when the birds could be transported by boat from the capture location to the experimental site. There were only a few times during the field season that this could be arranged.

At capture, the birds were ringed, measured and weighed (to nearest g). Fat score was determined using a 9-grade scale for visual fat classification developed by Pettersson and Hasselquist, (1985, grades 0–6) and extended at Falsterbo Bird Observatory (grades 7–9; Sjöberg et al., 2015). The age of the dunlins was identified on the basis of plumage characteristics (Prater et al., 1977). The birds were put into cardboard boxes after capture and transported either by float-plane or motorboat from the capture site to the field station. There, they were kept in a large white tent (Weatherport Inc., Delta, CO, USA) allowing the birds to experience the natural photoperiod and ambient temperature, but no outdoor celestial cues. Up to five dunlins were kept in the same spacious non-magnetic cage (50×100×50 cm) with unlimited food (mealworms and trout pellets) and water with vitamins. Individual dunlins were held in captivity for between 3 and 24 days and used in orientation experiments up to four times, but each individual is only represented once in each diagram. All dunlins were released at Kanaryarmiut Field Station after the experiments.

We used modified ‘Emlen funnels’ (Emlen and Emlen, 1966; Åkesson, 1994; Åkesson et al., 2005), lined with typewriter correction paper (Tipp-Ex, BIC GmbH, Eschborn, Germany; see Rabøl, 1979; Beck and Wiltschko, 1981) to record the dunlins’ migratory orientation. The orientation cages were made of non-magnetic materials (plastic bottom and sides, brass screws, plastic net topping), 18 cm high and with a top diameter of 60 cm, and the tops of the cages were covered with fine-mesh plastic netting allowing the birds to see approximately 160° of the natural sky overhead (Grönroos et al., 2010). The directional tendencies of the dunlins were recorded by analysing the distribution of scratches left by the birds’ claws on the pigment of the Tipp-Ex paper with a visual estimation method (see further above; Rabøl, 1979, 1995; Mouritsen, 1998; Åkesson et al., 2015).

Cloud cover was estimated visually in the beginning, middle and at the end of each experiment (0/8: cloudless; 8/8: completely overcast). We tested the directional preferences of the dunlins under both clear skies (1/8–6/8) and overcast conditions [natural overcast when cloud cover was 8/8 or simulated overcast under partly cloudy skies, with opaque diffusing Plexiglas sheets (3 mm) on top of the orientation cages]. Natural total overcast reduces the degree of polarization to 7% or less (Hegedüs et al., 2007), while simulated overcast using 3 mm Plexiglas sheets on top of the cages reduce the degree of polarization to <5% inside the cage (Åkesson et al., 2015). The motivation to express migratory activity is highly affected by the ability to see the sky and lead to higher proportion of the birds showing no migratory restlessness as compared to a clear sky situation (Åkesson, 1994). This reduction in activity is especially true for experiments under simulated overcast. In order to receive data from overcast conditions we performed tests under total overcast skies (8/8) without the Plexiglas sheets when those occasions were available, which was less than 30% of the tests under this condition.

The orientation cage experiments were carried out between 11 August and 19 September 2005, outdoors in a flat and open area without landmarks visible from within the orientation cages. Experiments lasted for 60 min and started within one and a half hours before local sunset, the time of day when many wader species normally initiate migration (Alerstam et al., 1990; Piersma et al., 1990; Gudmundsson, 1994; Hua et al., 2017), and when our dunlins were expected to depart on migration flights. If a bird was inactive during the test hour, it was tested again at another day until it showed activity. The elevation and azimuth of the sun in the middle of the experimental hour was calculated relative to geographic north by using a computer program developed by USNO Astronomical Applications Department (Washington, USA). Experiments were performed at sun elevations varying between 8° and −2° relative to the horizon.

### Data analysis and statistics

To determine the orientation of individual birds, as recorded by scratches from the claws in the pigment of the Tipp-Ex paper, we visually estimated the median direction to the nearest 5° (cf. Mouritsen and Larsen, 1998; see Grönroos et al., 2010 for details). The result of a given experiment was included only if at least 40 scratches were visible on the Tipp-Ex paper (see Table 1 for number of birds included in the experiments; Åkesson, 1994). Each paper was given a score between 0 and 4 for activity (0: <40 scratches, 4: >2000) and concentration (indicates the angle within which the mean direction without doubt lies; 0: >45°, 4: 0°–5°). Only if both scores had a value of at least one and if the sum of the two scores was at least three the result was included in further analyses. This ensured that disoriented and unreliable orientation results were excluded from further testing. Individual bearings were used to calculate a sample mean direction (*α*) and mean vector length (*r*) using vector addition according to standard procedures (Batschelet, 1981). The vector length (*r*) describes the scatter of the circular distribution (ranges between 0 and 1, the scatter being inversely related to the vector length). The Rayleigh test was used to test for significant directional preferences (Batschelet, 1981). Differences in mean angles between test categories was analysed by applying the one-way classification test (*F*_1,d.f._, Mardia, 1972). To compare the scatter around mean angles as given by the mean vector lengths we used the test for homogeneity of concentration parameters (*t*, Mardia, 1972). If one of the samples was randomly distributed, Watson's *U*^2^-test was used (Batschelet, 1981). Circular statistics tests were performed by programming in Microsoft Excel (Mardia's tests), and with Oriana version 4.01 (Rayleigh test, Watson's *U*^2^-test; https://www.kovcomp.co.uk; Pentraeth, UK). To analyse whether the mean orientation differed from the direction of the sunset point in the middle of the test hour or from the expected migratory courses or simulated route directions we used 95% confidence intervals (CI) according to Batschelet (1981).

Early caught dunlins were grouped according to fat score in one of two classes, ‘fat’ (fat score ≥3) or ‘lean’ (fat score <3) to check for any possible effect of body condition on the birds’ orientation performance where lean birds were expected to perform reverse migration and fat birds selecting directions towards the expected migratory direction (e.g. Lindström and Alerstam, 1986; Sandberg, 1994, 2003). It was not possible to group late caught dunlins in the two fat classes since there were too few fat birds.

### Route simulations

We simulated migratory routes from the experimental location Kanaryarmiut Field Station in the Yukon-Kuskokwim Delta assuming different route geometries produced by different vector navigation mechanisms given an initial geographic direction. In particular, we considered: (a) geographic loxodrome route, generated by a star compass (Emlen, 1975b), or a sun compass where the internal clock of the bird is constantly adjusted to the local time or a magnetic compass re-calibrated with celestial cues to adjust for the magnetic declination (e.g. Muheim et al., 2006); (b) time-compensated sun compass route, where birds select the direction relative to the sun azimuth at sunrise or sunset while keeping their internal clock set at the departure location (Alerstam and Pettersson, 1991); (c) magnetic loxodrome route, where direction is kept constant relative to the magnetic north; (d) magnetoclinic route, where the bird select its direction to keep a constant apparent geomagnetic inclination as in Kiepenheuer’ hypothesis (Kiepenheuer, 1984). For simplicity, we only included the sun compass mechanism that predicts the shortest route (i.e. orthodrome) and discuss alternative sun-based compasses (Muheim et al., 2018; Sokolovskis et al., 2019) below. Furthermore, we did not consider the effect of winds in our simulations (for more details on simulations see, Åkesson and Bianco, 2016, 2017). Route simulations, were performed assuming a length of 5000 km and final destinations either the Pacific Northwest or Japan/Korea (Fig. 1). Moreover, we performed two sets of simulations. The first set was used to investigate whether the initial geographic direction obtained from orientation experiments would result in a successful route for any of the four compass mechanisms. The second set of simulations was aimed at producing successful migratory routes by using a specific direction for each compass mechanism to be compatible with known sub-species’ wintering ranges. The second set of simulations allowed us to (1) verify that a vector navigation route would be theoretically possible for every compass mechanism and (2) how divergent predicted departing direction were between different compasses.

Simulations were done using R software ver. 3.4.4 (R Development Core Team, 2018) and the packages *geosphere* ver. 1.5-7, *lubridate* ver. 1.7.4 (Grolemund and Wickham, 2011), *raster* ver. 2.6-7 and *oce* ver. 0.9-23 (Kelley, 2018).
